# Mechanisms of transcriptional activation of the stimulator of interferon genes by transcription factors CREB and c-Myc

**DOI:** 10.18632/oncotarget.13183

**Published:** 2016-11-07

**Authors:** Yan-Yan Wang, Rui Jin, Guo-Ping Zhou, Hua-Guo Xu

**Affiliations:** ^1^ Department of Laboratory Medicine, The First Affiliated Hospital, Nanjing Medical University, Nanjing, Jiangsu Province 210029, China; ^2^ Department of Pediatrics, The First Affiliated Hospital, Nanjing Medical University, Nanjing, Jiangsu Province 210029, China; ^3^ Department of Pediatrics, Nanjing First Hospital, Nanjing Medical University, Nanjing, Jiangsu Province 210006, China

**Keywords:** stimulator of interferon genes, promoter, CREB, c-Myc

## Abstract

Stimulator of interferon genes (STING) plays an important role in host defense, autoimmune disease, osteoclast differentiation and anti-tumor response. Although many downstream targets have been studied in depth, the regulation of STING gene expression remains largely unknown. Here we demonstrate that transcription factors CREB and c-Myc maintain the transcriptional activity of STING. By 5′-rapid amplification of cDNA ends analysis, we identified the transcriptional start site (TSS) of STING. We illustrated that the region -124/+1 relative to TSS was sufficient for full promoter activity by a series of 5′ deletion promoter constructs. Transcriptional activity of the STING minimal promoter was dependent on CREB and c-Myc binding motifs and was abolished after mutation of these two DNA elements. Chromatin immunoprecipitation assays demonstrated that transcription factors CREB and c-Myc bind to STING promoter in vivo. Overexpression of CREB and c-Myc increased the STING promoter activity. Meanwhile, knocking-down of CREB and c-Myc by a small interfering RNA (siRNA) strategy markedly reduced endogenous STING expression. In summary, these results demonstrated that transcription factors CREB and c-Myc are involved in the regulation of STING transcription.

## INTRODUCTION

Stimulator of interferon genes (STING) (also known as MITA, MPYS, ERIS and TMEM173) [1–4], is a multispanning transmembrane protein that was originally identified as a growth inhibitor that mediates anti-MHC class II antibody-induced death in B lymphoma cells [5]. STING is expressed ubiquitously in multiple human tissues and cell lines and has been observed at the plasma membrane, endoplasmic reticulum (ER), and on the surface of the outer mitochondrial membrane [1, 2]. It is well known that STING controlled TLR-independent cytosolic responses to viruses and nucleic acids, being required for the activation of both IRF3 and NF-κB and for induction of the gene expressing IFN-β [6]. Recent evidence suggested that STING gets involved in STING-TBK1-IRF3 signaling pathway by three distinct cytosolic nucleic acid–sensing pathways, RNA sensing through RLRs, DNA detection by DDX41 and the ALR IFI16, and the sensing of bacterial cyclic dinucleotides, such as c-di-GMP (c[G(3’ -5’)pG(3’ -5’)p] or cyclic di-AMP or cyclic GAMP (cGMP–AMP) [2, 7–9]. Recent work has demonstrated that STING also plays an important role in antitumor response, autoimmune disease, and osteoclast differentiation [10–14]. STING is significantly downregulated in breast cancer patients as well as in ER positive breast cancer cell lines [15]. In addition, STING is a transcriptional target of E2 proteins of high risk HPV [16]. Activation of the STING pathway in tumor-resident host APCs is required for induction of a spontaneous CD8^+^ T cell response against tumor-derived antigens in vivo [7]. Furthermore, it contributes to the antitumor effect of radiation. It was recently reported that gain-of-function mutations in STING results in STING-associated vasculopathy with onset in infancy (SAVI), a syndrome characterized by a severe cutaneous vasculopathy leading to extensive tissue loss and structural damage, with neonatal-onset systemic inflammation [18–20]. Choe et al have demonstrated that the transcription factors c-Fos and NFATc1, two osteoclast-specific transcription factors, were down-regulated by overexpression of STING in RAW264.7 cells. In addition, they showed the induction of STING expression inhibited osteoclast differentiation by suppressing the RANKL-induced expression of c-Fos, NFATc1 and ERK activation [21].

Although functions and downstream targets have been studied in detail, cis elements or trans factors responsible for regulating STING gene expression remained largely unknown. In this study, we identified the minimal promoter of STING is located within the region -124/+1 bp relative to the transcription start site (TSS) and demonstrated that transcription factors CREB and c-Myc maintain the transcriptional activity of STING.

## RESULTS AND DISCUSSION

### Identification of the human STING transcription start site (TSS) and promoter region

The transcription start site of the human STING gene was identified by 5′ RACE analysis as described in the materials and methods. Three DNA fragments resulting from the 5’RACE nested PCR were obtained and products of three separate PCR reactions were cloned and sequenced. Direct sequencing of the nested PCR products indicated that the major transcription start site locates at 334 bp upstream from the ATG codon.

**Figure 1:**
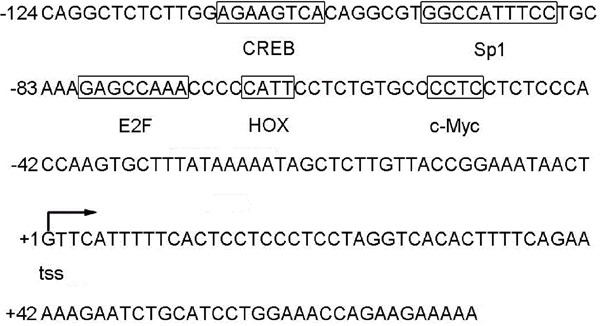
Nucleotide sequence of the human STING promoter Putative binding sites for transcription factors are indicated with boxes. The transcription start site is indicated by arrow.

To identify the human STING promoter, we employed Promoter 2.0 Prediction Program (http://www.cbs.dtu.dk/services/Promoter/) to search potential sequences. It showed a putative promoter locating within sequences near nucleotides -400 of the STING 5’ flanking region. Sequence analysis by using the TFSEARCH program (www.rwcp.or.jp/papia/) revealed that human STING promoter region contains several putative transcription factor-binding sites, such as CREB, Sp1, E2F, HOX and c-Myc (Figure 1).

### Identification of core region in the human STING promoter

**Figure 2:**
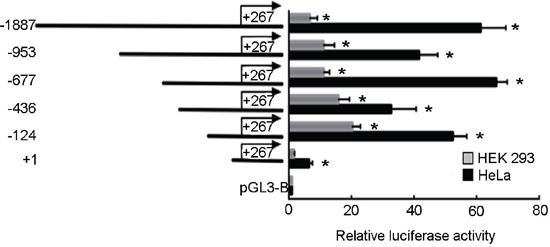
Deletion analysis of human STING promoter in HEK 293 and HeLa cells The human STING promoter sequence with different 5′ end (from −1887/+267 to +1/+267) was ligated to the firefly luciferase and inserted into plasmid pGL3 basic. The constructed vectors were transiently transfected into HEK 293 and HeLa cells and the luciferase activities were measured. Each transfection reaction in an assay was carried out in triplicate and assays were repeated three times. Values were normalized by co-transfecting Renilla luciferase reporter gene. Data (means ± standard deviation) were expressed as fold over vehicle treated control, which was arbitrarily set as 1. (*)*P* < 0.05 calculated with Student's t-test.

To determine the regulatory element of human STING gene, we cloned the 2154 bp fragment (-1887 to +267 relative to TSS) into the promoterless pGL3-Basic luciferase reporter plasmid and designated the plasmid pGL-1887. Then the plasmid pGL-1887 was transiently transfected into HEK 293 and HeLa cells. As shown in Figure 2, it expressed high level of luciferase activity (60-fold more than pGL3-Basic) in HeLa cells, indicating that it contains human STING promoter region.

To locate the minimal sequence responsible for the transcription of the human STING gene, a series of deletion constructs were performed by inserting different fragments of the human STING promoter with a fixed 3′ end at the +267 bp position (relative to TSS) but a different 5′ end into the pGL3-Basic vector and then were transiently transfected into HEK 293 and HeLa cells. Luciferase assays revealed pGL3+1 have little promoter activity. Much stronger promoter activities were observed in pGL3-124, 436, 677, 953 and 1887 (Figure 2). These results indicate that the sequences between -124 and +1 are sufficient for eliciting the basal transcription of STING.

### Functional analysis of putative binding sites for CREB and c-Myc in human STING promoter

**Figure 3:**
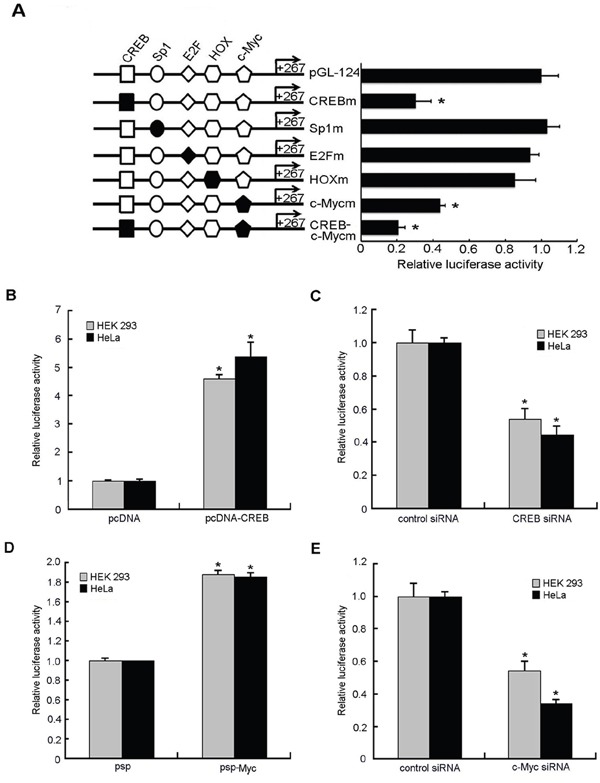
Effects of putative transcription factors on human STING promoter activity **A.** Mutation analysis of the transcription factors binding sites in the human STING promoter. A series of mutants were constructed and used for transient promoter assays in HEK 293 and HeLa cells. The reporter activity was expressed as the fold increases in relative promoter activities compared with that in the pGL-124. **B, D.** HEK 293 and HeLa cells were cotransfected with either plasmids pcDNA/psp (300ng) or CREB/c-Myc overexpression vectors pcDNA-CREB/psp-Myc(300ng) and STING reporter plasmids(100ng), renilla luciferase plasmid pRL-TK (2ng). Relative luciferase activity of the STING promoter controlled by samples treated with empty vectors pcDNA or psp was set as 1. **C, E.** STING reporter plasmid(100ng) and pRL-TK (2ng) together with CREB siRNA, c-Myc siRNA or negative control siRNA (50nM) were transfected into HEK 293 and HeLa cells individually. Relative luciferase activity of the STING promoter controlled by samples treated with negative control siRNA was set as 1. The data for all were normalized for Renilla luciferase activity and means and standard deviations of three independent experiments, with each experiment carried out in triplicate. (*)*P* < 0.05 calculated with Student's t-test.

A search for transcription factor binding sites by using online software (TFSEARCH) revealed the presence of several putative binding sites, including CREB, Sp1, E2F, HOX and c-Myc, in the promoter region of STING gene. In order to evaluate the contribution of the putative binding sites to the regulation of the STING promoter, we made point mutations of them as described above in the context of the reporter construct, and these constructs were subjected to reporter gene assays in comparison to the wild-type construct. Mutation of the CREB and c-Myc sites reduced STING promoter activity to 31% and 43% of the wild-type promoter activity, respectively. Double CREB and c-Myc mutation reduced the promoter activity to 21% of the wild-type promoter activity (Figure 3A). However, single mutation of Sp1, E2F or HOX had little effect on promoter activity. These results indicate that the CREB and c-Myc sites play important roles in basal transcription of STING.

In order to confirm the critical regulatory function of CREB and c-Myc sites, transcription factors CREB and c-Myc were overexpressed. The results (Figure 3B, 3D) showed that overexpression of CREB and c-Myc increased the activity of STING promoter by 5 times and 1.8 times, respectively.

To further confirm the role of CREB and c-Myc in the regulation of STING promoter activity, we used RNA interference to knock down CREB or c-Myc expression in HEK 293 cells and HeLa cells. We cotransfected STING reporter plasmid together with CREB siRNA, c-Myc siRNA or negative control siRNA into HEK 293 cells and HeLa cells, respectively. The results showed a 50~70% reduction of the luciferase activity in the presence of CREB- or c-Myc-Specific siRNA compared to that with negative control siRNA (Figure 3C, 3D). In addition, functionality of the siRNA directing against CREB or c-Myc and their overexpression plasmids was confirmed by RT-qPCR and western blot analysis: they were effective on the CREB or c-Myc mRNA and protein levels (Figure 5C–5F).

Based on the above experimental results, we conclude that CREB and c-Myc positively regulate the basal transcriptional activity of STING.

### CREB and c-Myc bind to STING promoter region in living cells

**Figure 4:**
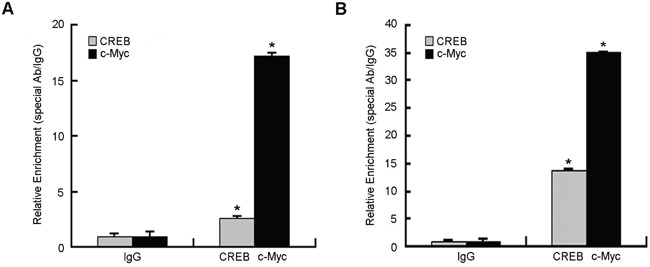
Chromatin immunoprecipitation assay (ChIP) of STING promoter was performed in HeLa cells **A.** Binding of transcription factors CREB and c-Myc to STING gene promoter in HeLa cells without any treatment. **B.** HeLa cells were harvested at 48 h after transfected with overexpression plasmid of CREB and c-Myc and then performed ChIP assay. The immunoprecipitated chromatin fragments for (A and C) were analyzed by quantitative PCR using primer pairs spanning the putative CREB, c-Myc binding site as described in Materials and Methods. Data were expressed as relative fold increase of specific antibody over normal IgG control. Each experiment was performed triplicate and (*)*P* < 0.05 calculated with Student's t-test.

To determine whether CREB and c-Myc bind to the STING promoter region, we performed chromatin immunoprecipitation (ChIP) experiments on HeLa cells. CREB- and c-Myc-associated DNA fragments were immunoprecipated using an antibody that recognizes the CREB or c-Myc subunit, respectively. Normal rabbit IgG was used as a negative control. DNA fragments were then amplified by real-time quantitative PCR using primers surrounding the potential binding sites for CREB and c-Myc. Figure 4A showed that anti-CREB and anti-c-Myc antibodies precipitated proteins bound in vivo to the amplified sequence of the STING promoter region whereas non-specific IgG (control antibody) failed to precipitate proteins bound in vivo this sequence. We also performed another ChIP assay in CERB and c-Myc overexpressing Hela cells. The results (Figure 4B) showed that overexpression of CREB and c-Myc obviously increased the anti-CREB and anti-c-Myc antibodies precipitated proteins bound in vivo to STING promoter. These data suggest that CREB and c-Myc can bind to the STING promoter region in vivo.

### CREB and c-Myc positively regulate the expression of STING

**Figure 5:**
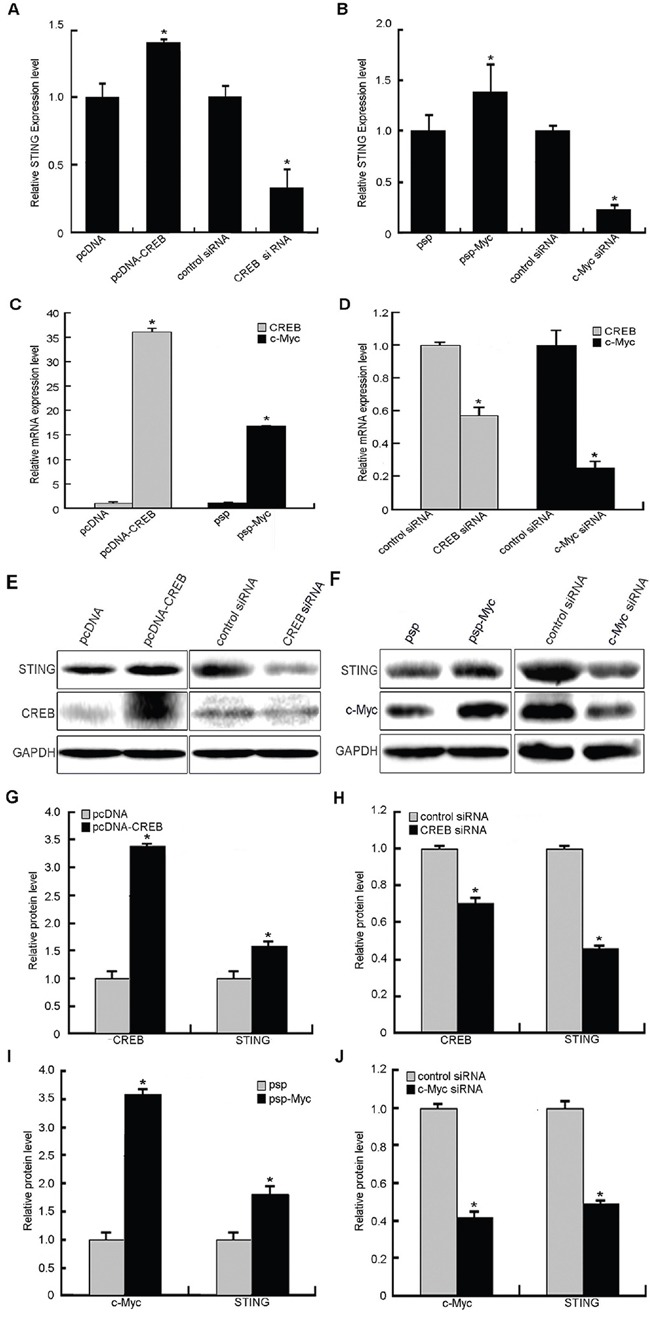
Effects of transcription factors CREB and c-Myc on human STING expression levels **A, E.** STING expression levels (mRNA and protein) were significantly increased or reduced after CREB overexpression or siRNA, respectively. **B, F.** Overexpression or siRNA of c-Myc caused increase or reduction of STING on its mRNA and protein levels. **C, D, E, F.** Increased and reduced CREB or c-Myc expression on mRNA and protein levels following CREB or c-Myc overexpression and siRNA interference. **G, H, I, J.** Results of protein gray scanning based on the pictures E and F. Each experiment was performed triplicate and (*)*P* < 0.05 calculated with Student's t-test.

To examine whether CREB and c-Myc affected the expression of STING, the mRNA and protein levels of STING were determined in HeLa cells after transient transfection with CREB or c-Myc expression vector or siRNA against CREB or c-Myc by real-time quantitative RT-PCR and western blot analysis. The results (Figure 5) showed that the mRNA and protein levels of STING were increased 1.3~1.4-fold and 1.4~1.9-fold when CREB or c-Myc were overexpressed, respectively. Meanwhile, the use of siRNA against CREB or c-Myc caused 75~80% and 40~45% reduction of STING mRNA and protein expression compared to negative control, respectively. These data demonstrate that CREB and c-Myc positively regulate expression of the endogenous STING gene at mRNA and protein levels.

It was reported that transcription factor CREB can cause repression of HCMV replication and may contribute to the development of new anti-HCMV strategies [22]. The features of CREB in Hodgkin lymphoma (HL) strongly support its role as a tumor suppressor gene that can decelerate cell proliferation by inhibiting the expression of several cell cycle-related genes [23]. In this study, we revealed that transcription factors CREB and c-Myc positively regulated human STING gene promoter. Considering that STING plays an important role in antiviral and anti-tumor response, our results further indicated that the antiviral and anti-tumor activities of CREB. This study can serve as a basis for future studies aimed at understanding how human STING gene expression is controlled in different tissues and how these transcription factors can be used to develop novel therapeutic strategies against viral infection and tumor.

## MATERIALS AND METHODS

### Cell culture

Human embryonic kidney (HEK) 293 cells and Human cervical cancer (HeLa) cells were obtained firstly from the American Type Culture Collection (ATCC). The cells were cultured in Dulbecco's modified Eagle's medium (DMEM) with 10% fetal bovine serum (FBS), 100unit/ml penicillin and 100 mg/ml streptomycin at 37°C in an atmosphere of 5% CO_2_.

### RNA analysis by 5′-rapid amplification of cDNA ends (5′RACE)

Cellular mRNA was prepared from HeLa cells using Trizol reagent followed by chloroform-isopropanol extraction and ethanol precipitation. 5′RACE was performed using a 5′/3′RACE Kit (Roche), according to the manufacturer's instructions. Three antisense STING gene-specific primers were needed for the first-strand cDNA synthesis and nested PCRs, including 5′-GGTCAGCCATACTCAGGTTATCAGG-3′, 5′-CCACAGTCCAATGGGAGGAGAAT-3′, 5′-ACCCCGTAGCAGGTTGTTGTAA TG-3′. Products from the nested PCRs were electrophoresed in a 1.2% agarose gel, purified, and then sequenced.

### Cloning of human STING promoter region and construction of pGL3 luciferase reporter plasmids

A 2154 bp 5′-flanking region of human STING gene was amplified by polymerase chain reaction (PCR) and then was cloned into the *Kpn* I and *Bgl* II sites of pGL3-Basic (Promega). The forward primer was: 5′-CGG*GGTACC*AACCCAGCCTCG CCCGTCTGTCTA-3′ (a restriction site for *Kpn*I underlined). The reverse primer was: 5′-GGA*AGATCT*AGCCTCCATTCCATTGCCCTTTG-3′ (a restriction site for *Bgl*II underlined). Similarly, the 5′ deletion clones were constructed with PCR by the above reverse primer, which was paired with the following forward primers:

(-953 to/+267: 5′-CGG*GGTACC*TGGGACTATAGGCGTGTTCCGTC-3′);
(-677 to+267: 5′-CGG*GGTACC*GGGCTAGAATTATACCTACCTGA-3′);
(-436 to+267: 5′-CGG*GGTACC*TGGAGTGCAGTGGCCTAATCTCT-3′);
(-124 to +267: 5′-CGG*GGTACC*CAGGCTCTCTTGGAGAAGTCACA-3′);
(+1 to +267: 5′-CGG*GGTACC*GTTCATTTTTCACTCCTCCCTCC-3′); DNA sequence analysis confirmed these sequences.

### Plasmids and siRNAs

The amplified promoter fragments were cloned into the *Kpn* I and *Bgl* II sites of pGL3-Basic (Promega). The expression plasmids CREB, pcDNA empty vector were stored by our group. The expression plasmids psp-myc, psp empty vector were kindly provided by Dr. Therése Wahlströom. CREB siRNA, c-Myc siRNA and the negative control were designed and synthesized in Genepharma company (Shanghai, China). Sequences targeted in the CREB and c-Myc mRNA, as well as the negative control sequence were listed below:

CREBi: 5′-AGUAAAGGUCCUUAAGUGCTT-3′
c-Myci: 5′-GGUGAUCCAGACUCUGACCUU-3′
control: 5′-UUCUCCGAACGUGUCACGUTT-3′

### Site-directed mutagenesis

Mutations of the CREB and c-Myc binding sites found in the STING promoter were performed using the QuikChange Site-directed Mutagenesis kit (Takara) according to the manufacturer's instructions. Oligonucleotides with site-specific mutations at the critical nucleotides necessary for transcription factor were listed in Table 1. The mutations were confirmed by sequencing.

**Table 1:** Sequences of oligonucleotides used in site-directed mutagenesis

Name	Sequence
CREBm F	GGCCACGCCTGT**TCG**TTCTCCAAGAGA
CREBm R	ATTTCCTGCAAAGAGCCAAACCCCCATTC
Sp1m F	GAAATGGCCA**GAA**CTGTGACTTCTCCAAGA
Sp1m R	CTGCAAAGAGCCAAACCCCCATTCCTCT
E2Fm F	CCCCCATTCCTCTGTGCCCCTCCTCT
E2Fm R	TTTGCAGGAAATGGCCACGCCTGTGACTT
HOXm F	GGGGTTTGGCTCTTTGCAGGAAATGGCCA
HOXm R	TCTGTGCCCCTCCTCTCCCACCAAGTG
c-Mycm F	CTCTCCCACCAAGTGCTTTATAAAAATAGCTC
c-Mycm R	GGCACAGAGGAATGGGGGTTTGGCT

The bold italics indicated the mutation sites

### Transient transfections and dual-luciferase reporter assays

Transfections were carried out in HEK 293 cells and HeLa cells by using Lipofectamine-2000 transfection reagent (Invitrogen) according to the manufacturer's suggestion. Cells having been seeded into 48-well plates 24 h before transfection were cotransfected with 400ng of each of the luciferase containing plasmids together with 4ng of a control pRL-TK plasmid as an internal control. After 48 h of transfection, cells were harvested and Luciferase assay was performed by using the Dual Reporter assay system (Promega) and TD-20/20 Turner Designs luminometer according to the manufacturer's instruction. Results were representative of at least three independent experiments performed in triplicate. For overexpression or RNAi, the expression plasmid (300ng) or siRNA (50nM) was individually cotransfected into HEK 293 cells and HeLa cells, together with STING promoter reporter plasmids (100ng) by using Lipofectamine-2000. Luciferase assay was performed 48 h after transfection.

### Chromatin immunoprecipitation assay

The chromatin immunoprecipitation assay was performed by using the Magna ChIP™ kit (Millipore) following the manufacturer's instructions. Briefly, 1×10^7^ subconfluent HeLa cells were fixed with 1% formaldehyde for 10 min at room temperature. After fixation, cells were washed twice in ice-cold PBS and harvested by centrifugation at 4°C. The cell pellets were resuspended in 500μl nuclear lysis buffer and sonicated using six 15 sec pulses with 50 second rest in between pulses at 5% of maximum output strength on a Sonicator Ultrasonic Processor (Qsonica, LLC). The antibody used in the ChIP assays was an anti-CREB antibody (Abcam), an anti-c-Myc antibody (Abcam) and rabbit IgG control antibody (Millipore) was used as a negative control. ChIP purified DNA was amplified by quantitative PCR (Q-PCR). The ChIP primers were as follows: forward 5′-GCTCCTACCTAATATCATCCCC-3′ and reverse 5′-AGTTATTTCCGGTAACAAGAGC-3′. Q-PCR was performed in an ABI 7300 real time PCR system (Applied Biosystems Inc.) and SYBR Green was used as fluorescent dye with each sample loaded in triplicate. A comparative *Ct* method (ΔΔ*Ct*) was used to calculate the relative fold enrichment of the immunoprecipitated DNA in all samples.

### RNA purification and quantitative real-time RT-PCR

Total RNA extraction was performed by using Trizol reagent followed by chloroform-isopropanol extraction and ethanol precipitation. Subsequently, duplicate samples of 2μl of each cDNA were used as a template. The quantification of gene transcripts was performed by real-time PCR using SYBR green I dye (Takara) and the ABI 7300 real time PCR system (Applied Biosystems Inc.). The specificity of amplification was assessed for each sample by melting curve analysis. Expression values were normalized with control GAPDH. The primers used are as follows:

CREB: forward 5′-CATTAACCATGACCAATGCAG-3′,
reverse 5′-CTGTGCGAATCTGGTATGTTT-3′;
c-Myc: forward 5′-CACCAGCAGCGACTCTGA-3′,
reverse 5′-GATCCAGACTCTGACCTTTTGC-3′;
STING: forward 5′-GGGCTGGCATGGTCATATTA-3′,
reverse 5′-TACTCAGGTTATCAGGCACC-3′;
GAPDH: forward 5′-GTCAACGGATTTGGTCTGTATT-3′,
reverse 5′-AGTCTTCTGGGTGGCAGTGAT-3′.

### Western blot analysis

All samples were lysed in Laemmli buffer, boiled, and electrophoresed on SDS-polyacrylamide gel, then separated proteins were transferred onto polyvinylidene difluoride membranes. To block non-special sites, the membranes were incubated in 5% dry milk in TBS-T saline (0.25 M Tris-HCl pH 7.6, 0.19 M NaCl, 0.1% Tween 20) for 1 h and then protein blots were incubated for overnight at 4°C with primary antibodies against GAPDH (Santa Cruz Biotechnology), CREB, c-Myc, STING (all from Abcam). Membranes were washed twice with TBS-T and treated with either a horseradish peroxidase-linked goat anti-mouse or anti-rabbit IgG (Santa Cruz Biotechnology). Reactive proteins were visualized by enhanced chemiluminescence (Cell Signaling Technology, Inc).

### Statistical analysis

The results in the present were analyzed using paired two tailed student's-t test. A *p* value ≤0.05 was considered statistically significant. Data are presented as the means±SD and all experiments were done in triplicate and repeated at least three times.
